# BioWarehouse: a bioinformatics database warehouse toolkit

**DOI:** 10.1186/1471-2105-7-170

**Published:** 2006-03-23

**Authors:** Thomas J Lee, Yannick Pouliot, Valerie Wagner, Priyanka Gupta, David WJ Stringer-Calvert, Jessica D Tenenbaum, Peter D Karp

**Affiliations:** 1 Bioinformatics Research Group, SRI International, Menlo Park, USA; 2 Computer Science Laboratory, SRI International, Menlo Park, USA; 3 Stanford Medical Informatics, Stanford University, Stanford, USA

## Abstract

**Background:**

This article addresses the problem of interoperation of heterogeneous bioinformatics databases.

**Results:**

We introduce BioWarehouse, an open source toolkit for constructing bioinformatics database warehouses using the MySQL and Oracle relational database managers. BioWarehouse integrates its component databases into a common representational framework within a single database management system, thus enabling multi-database queries using the Structured Query Language (SQL) but also facilitating a variety of database integration tasks such as comparative analysis and data mining. BioWarehouse currently supports the integration of a pathway-centric set of databases including ENZYME, KEGG, and BioCyc, and in addition the UniProt, GenBank, NCBI Taxonomy, and CMR databases, and the Gene Ontology. Loader tools, written in the C and JAVA languages, parse and load these databases into a relational database schema. The loaders also apply a degree of semantic normalization to their respective source data, decreasing semantic heterogeneity. The schema supports the following bioinformatics datatypes: chemical compounds, biochemical reactions, metabolic pathways, proteins, genes, nucleic acid sequences, features on protein and nucleic-acid sequences, organisms, organism taxonomies, and controlled vocabularies. As an application example, we applied BioWarehouse to determine the fraction of biochemically characterized enzyme activities for which no sequences exist in the public sequence databases. The answer is that no sequence exists for 36% of enzyme activities for which EC numbers have been assigned. These gaps in sequence data significantly limit the accuracy of genome annotation and metabolic pathway prediction, and are a barrier for metabolic engineering. Complex queries of this type provide examples of the value of the data warehousing approach to bioinformatics research.

**Conclusion:**

BioWarehouse embodies significant progress on the database integration problem for bioinformatics.

## Background

The importance of the database (DB) integration problem to bioinformatics has been recognized for many years [1-6]. Many questions in bioinformatics can be addressed only by combining data from multiple DBs, and DB integration also permits cross-checking of individual DBs for validation. In 1993 a summary of a U.S. Department of Energy workshop on genome informatics noted that "achieving coordination and interoperability among genome DBs and other informatics systems must be of the highest priority. ... We must begin to think of the computational infrastructure of genome research as a federated information infrastructure of interlocking pieces..." [7].

One approach has involved mediator-based solutions that transmit multidatabase queries to multiple source DBs across the Internet. Although some progress has been made in developing mediator technology, we argue that these systems face several practical limitations (see Section "Comparison of the Warehouse and Multidatabase Approaches" for more details), including that (a) few source DBs accept complex queries via the Internet (an immediate deal killer), (b) the user lacks control over which version of the data is queried, and over the hardware that provides query processing power, (c) the speed of the Internet limits transmission of query results, and (d) users cannot cleanse the source DBs that they query of potentially erroneous, incomplete or redundant data – that is, they cannot alter the source DBs in any way. The DB warehouse approach overcomes all of these limitations.

This paper describes an evolving open source toolkit for constructing DB warehouses that combines different collections of bioinformatics DBs within a single physical DB management system (DBMS) to facilitate queries that span multiple DBs. We emphasize that BioWarehouse is a flexible toolkit that supports multiple alternative warehouses: our goal is to enable different investigators to create different warehouse instances that combine collections of DBs relevant to their interests. The warehouse also facilitates integration of locally produced data with other public bioinformatics DBs in pursuit of goals such as capture of experimental data, sharing experimental data with collaborators, Internet publishing of data to the scientific community, and data mining and global integrative studies across multiple DBs. The data sources supported by BioWarehouse are particularly well suited to integration of pathway information, although pathways are only one of many datatypes supported by BioWarehouse.

SRI operates a publicly accessible BioWarehouse server called Publichouse that contains four of the DBs supported by BioWarehouse. See URL [8] to obtain an account.

### Motivations

The 2004 online compilation of molecular biology DBs prepared by *Nucleic Acids Research *lists approximately 580 DBs [9]. These DBs represent a significant investment whose full potential has not been realized due to integration barriers. This is due to the need of different scientific projects to access information from multiple different DBs to meet their objectives. As bioinformatics DBs grow in size, and as biological questions grow in scope, the point-and-click style of DB mining becomes less and less practical in such an environment. Instead, computational biologists seek discoveries by using programs and complex queries to mine DBs. These programs seek new patterns and generalizations by issuing queries to one or more DBs to select, combine, and compute over millions of data records. Unfortunately, many barriers exist to the querying of multiple DBs, including mismatches in query language, access mechanisms, data models, and semantics.

Combining information from multiple DBs is important for two principal reasons. First, information about a given biological entity is often scattered across many different DBs: the nucleotide sequence of a gene may be stored in one DB, the three-dimensional structure of its product may be stored in a second DB, information about the expression of the gene may be stored in a third DB, and data regarding interactions of the gene product with other proteins may be stored in a fourth DB. Bioinformatics data tends to be organized around a given type of experiment (such as gene expression or protein structure determination), yet many types of scientific investigations require combining data from different experiment types. For example, one way to partially validate whether an observed protein-protein interaction is of physiological relevance is to ask whether the genes for both proteins are expressed in the same cell type; answering this question requires combining information from multiple DBs.

Second, DB integration is important because different DBs often contain redundant or overlapping information. DB integration allows cross-validation and verification of the DBs to identify such information.

**Figure 1:**
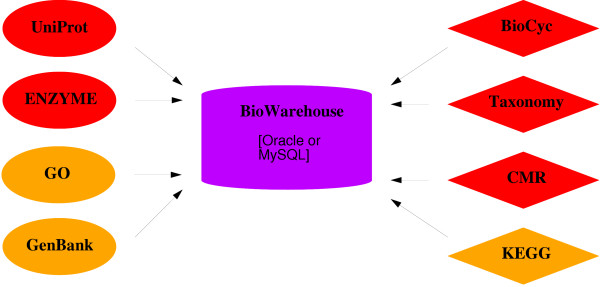
**Architecture of the BioWarehouse system.** Loader tools parse source databases and insert their contents into BioWarehouse. Oval shaped loaders are written in Java, diamond shaped loaders are written in C.

In summary, advances in biology are hindered not by lack of data, but by the diversity of technologies used for storing the data. Here we propose a solution, as shown in Figure 1, whereby the DBs required for a project (or set of projects) are collected into a single high-performance environment operating from a local server so that scientists can control the set of DBs, the version of each of these DBs, and the performance of the overall system. This approach also enables scientists to combine data they have produced locally with data from public bioinformatics DBs.

### Comparison of the warehouse and multidatabase approaches

We define the multidatabase approach (also known as the federated approach) to DB integration as the approach whereby users employ a special-purpose query language to formulate a single query that spans multiple DBs [10]. A mediator software system then dissects the query into subqueries relevant to individual DBs, transmits the subqueries via the Internet to each DB, combines the results of the subqueries, and returns the result to the user. Systems that employ the multidatabase approach include K2 [11] developed at the University of Pennsylvania, BioKRIS [12], OPM [13], TAMBIS [14], and BioMediator [15].

This approach has two potential advantages over the warehouse approach. First, it allows users to avoid investing in the hardware required to replicate the source DBs locally. The significance of this barrier depends, of course, on the number and size of the DBs to be integrated. Second, it provides users with immediate access to the freshest version of each source DB. However, warehouses can provide fresh data if they are updated frequently. Yet, refreshing large DBs can involve frequent downloads that are costly in network bandwidth, unless the DB offers downloads of changed entries only (deltas). Very few public bioinformatics offer delta downloads, which we argue should be an area for future research, to decrease the costs of warehouse updating.

However, several limitations are associated with the mediator approach:

• A key limitation of the multidatabase approach is that it rests on a faulty assumption, namely, that all the bioinformatics DBs that users want to query are queryable via the Internet by using a network query API with the expressive power of a language such as SQL.

That is, the assumption is that the multidatabase approach will allow us to integrate DBs that are ready and waiting in a queryable form. However, the vast majority of bioinformatics DBs have not been made queryable in this manner by their developers, and this situation has changed little in the past decade. The DiscoveryLink system [16] does provide technology for wrapping into an SQL-queryable from data sources that are available as files. We expect that writing such a wrapper would be similar in complexity to writing a BioWarehouse DB loader. Furthermore, once wrapped in this manner, some advantages of the multidatabase approach (such as instant access to newly released versions of the data) are lost.

• The performance of the Internet limits query speed because potentially large intermediate results must be sent across relatively slow Internet links.

• Every source DB server that is accessed by a user query must be available during execution of that query; the more sources a query accesses, the higher the probability that at least one source will be unavailable.

• The fact that users do not control the hardware on which queries submitted to a mediator are run can be problematical for users whose applications produce large numbers of queries and/or produce large query results. It is unrealistic to expect a single Internet- accessible server for a given public DB to be able to satisfy the computational demands of the entire biomedical research community, and mediator users have no control over the hardware supporting the DBMS servers to which their queries are sent. This problem can be solved by installing a local warehouse whose hardware is configured to satisfy the query processing demands of the user's application (e.g., a large-memory multiprocessor).

• Although access to up-to-date data can be an advantage of the multidatabase approach, it can also be a disadvantage. Some users need control over what dataset version they are querying, and do not want that version changing out from under them. For example, a user who is running a software package that functions with version 3.0 of a DB, but that breaks under version 4.0 of that DB, wants control over when to migrate to version 4.0. Similarly, a user who is training and evaluating a machine learning program may want to perform experiments with respect to a constant version of the DB for consistency in evaluating program performance.

• Mediator users are unable to perform cleansing of remotely accessed DBs. In contrast, DBs loaded into a local warehouse can be cleansed of what a user considers to be erroneous or noncompliant data. If desired, this extracted data can then be stored as a distinct dataset within the warehouse.

In addition, users who generate their own local experimental or computationally derived datasets need a DBMS in which to house them. The warehouse fulfills that need, whereas the multidatabase approach is unable to store locally produced data.

Taking full advantage of the BioWarehouse and its preceding benefits does not preclude integration of databases not yet supported by the BioWarehouse. BioWarehouse may be used in a complementary fashion with a mediator system in that a multidatabase query engine can sit above BioWarehouse and enable access to additional DBs. This software can then send subqueries to BioWarehouse when it contains the relevant data, whereas other subqueries can be sent to external Internet-accessible DBs.

In summary, our intention is not to rule out use of the mediator approach, but to present what we see as a systematic discussion of the strengths and weaknesses of both approaches, many of which have not been discussed previously.

### Definitions

A **data object **is an element of a description of a biological system at a particular granularity and with respect to a particular ontology. For example, a data object might correspond to a gene, or to a control region within a gene, or to a protein-protein interaction. A **database **(DB) is a collection of data objects. For example, Swiss-Prot is a DB. A **dataset **is a specific version of a DB. For example, Swiss-Prot version 39.0 is a dataset.

A **database management system **(DBMS) is a software system for storing and querying collections of data. A **data model **is the primitive data structuring mechanism used by a DBMS, such as the relational data model used by relational DBMSs. A **schema **is the set of all tables used to represent data objects. A **subschema **is a set of related DB tables within the warehouse schema, such as all tables involved in defining the representation of a data object.

A **BioWarehouse instance **results from integrating a specific set of datasets into the BioWarehouse schema within a single DBMS. An instance could contain multiple versions of the same DB. A **warehouse object **is the representation of a data object in a warehouse. A **warehouse identifier **(WID) is an integer unique identifier assigned within a BioWarehouse instance to a warehouse object.

## Implementation

Design requirements of BioWarehouse reflect goals of simplicity, accessibility, efficiency, and scientific utility. This section discusses these requirements, and the corresponding design decisions that were made to satisfy them.

### Overall requirements

The warehouse must scale to allow effective operation with terabytes of data. Each dataset is expected to be gigabytes in size, and available datasets typically increase significantly in size each year. **Decision: **Relational DBMS technology is in much more prevalent use in the bioinformatics community than is object-oriented DBMS technology, and has better scalability; therefore, BioWarehouse uses relational technology.

The warehouse should be compatible with standard freeware DBMSs and commercial-grade DBMSs that are most commonly used in the bioinformatics community, to allow users who cannot afford the cost of commercial DBMSs to employ freeware DBMSs, and to allow users who cannot afford the limitations of freeware DBMSs to employ commercial DBMSs. **Decision: **BioWarehouse supports the Oracle and MySQL DBMSs.

The BioWarehouse architecture should be scalable to support the integration of a growing number of data sources. **Decisions: **The project is open source to permit contributions of loaders from many groups. In addition, the complexity of the schema must be constrained to facilitate extensibility (see next section).

Multiple access mechanisms must eventually be provided including SQL, XML-based methods, Open Agent Architecture (OAA) [17], CORBA, and Web-based query interfaces. **Decision: **Initial support is provided for SQL (because of its pervasiveness) and for OAA (because of its use in the DARPA Bio-SPICE program).

For installation, given an operating DBMS the requirements for the person configuring a warehouse instance are limited to basic DB administrator (DBA) expertise.

### Schema requirements

The BioWarehouse schema should be as simple as possible. If its schema grows too large, that complexity could be a significant barrier to widespread adoption of the warehouse technology, because users will find the schema so difficult to understand that they will be unable to write queries or application programs. Furthermore, because its schema must evolve as BioWarehouse grows to support new datatypes, a complex schema will be a significant barrier to the further development of BioWarehouse, both at SRI and by collaborators at other institutions, thus limiting the scalability of BioWarehouse to more data sources.

**Decisions: **We define single common tables for information that is common to many warehouse datatypes, to decrease the schema complexity. For example, comments and citations are common to many warehouse datatypes (such as to genes, proteins, and pathways), and each is implemented through a single table. We must, of course, define an association between a comment and a warehouse object. If different BioWarehouse object types used different spaces of object identifiers, uniquely defining that association would require the schema to encode both the object ID and the object type, since objects of different types could have the same ID. Thus, we use a simpler approach whereby all BioWarehouse objects share a single space of object identifiers within a warehouse instance. The identifiers are known as warehouse identifiers (WIDs), and are integers that are assigned at DB load time. The use of a single space of WIDs allows associations between, for example, a comment and an object, to specify a WID only, and not the object type also.

The warehouse schema must support the concurrent presence, accessibility, and addressability of multiple datasets (multiple versions of a given DB) within a BioWarehouse instance.

The warehouse schema should facilitate coercion of different sources of the same type of data into a common semantic framework. For example, a warehouse might be created that contains both the Swiss-Prot and PIR protein DBs (note that UniProt does not contain all information from PIR). Once loaded, the two DBs would exist side by side within the warehouse, without having been merged, but within the common warehouse schema. A separate project within the same BioWarehouse instance could create yet a third dataset within the warehouse consisting of a nonredundant merging of Swiss-Prot and PIR. This approach implies that the warehouse user need learn only the schema of the warehouse, not of each data source, whereas some mediator systems lack a global schema and require the user to know the schema of each DB that they query.

### Loader requirements

Because bioinformatics DBs are often large and may have a relatively poorly defined syntax, load failures are frequently observed and without precautions could result in crashing the loader. For this reason, DB loaders should be able to recover gracefully from errors encountered during parsing of their input files. **Decision: **The loaders are designed to keep loading even in the presence of an error. If partial data has been inserted into the warehouse, a LoadError flag maintained on the related objects is updated to indicate that an error occurred while parsing the object, and that the warehouse entry for the object may therefore be incomplete or contain errors.

### BioWarehouse schema design

We designed the BioWarehouse schema by first studying the schemas of each DB to be integrated, as well as the schema of other DBs that use the same datatype. Our experience in developing the Pathway Tools ontology [18], which spans many warehouse datatypes, was also helpful.

The development of the BioWarehouse schema was guided by several principles, illustrated in this example involving Swiss-Prot, TrEMBL, PIR, and EcoCyc. Although the exact fields present in these DBs vary, all of them contain information about proteins; therefore, we consider the protein subsets of these DBs to be a single datatype.

Since DBs typically conceptualize proteins in different ways, any kind of cross-DB operation faces the problem of semantic heterogeneity, whereby information is partitioned in different fields that use different definitions (such as different units of measure). One possible approach to supporting protein DBs within the warehouse would be to create different schema definitions for each of the conceptualizations of proteins used by the source DBs. However, this approach would perpetuate the semantic heterogeneity among these DBs, and would complicate the resulting schema of BioWarehouse. At the extreme, BioWarehouse would have to contain a different protein schema for every protein DB that it loads. Therefore, the warehouse schema would be larger and more complex, and users would have more difficulty understanding the schema, making it more difficult for a user to query the DB. Under this approach, a user who wanted to query all proteins in the DB would have to study the subschema for each different protein DB in the warehouse, and essentially write a separate subquery for each subschema.

Instead, the warehouse approach uses a single set of schema definitions to cover a given datatype, even if that datatype is conceptualized differently in different DBs. For example, we create a single set of schema definitions to span all attributes for proteins. The DB loaders are responsible for translating from the conceptualization used in each DB within the family to the conceptualization used by the BioWarehouse schema. This approach eliminates the semantic heterogeneity of these DBs, allowing users to query all protein sequence DBs using the same schema.

Another important element of our approach is to explicitly encode the dataset from which each data object within the warehouse is derived. For example, since entries from any protein DBs are loaded into the same set of tables, it is critical for user queries to be able to distinguish Swiss-Prot entries from TrEMBL entries. Thus, queries can request the retrieval of all warehouse protein sequences that were loaded from the Swiss-Prot dataset.

Our DB loaders do not attempt to remove redundancy during the loading of multiple DBs, so if Swiss-Prot and PIR were loaded, and contained entries describing the same protein, two distinct entries would be created in the warehouse. This is important for four reasons: (1) Scientifically, we believe that the warehouse should respect and maintain the integrity of individual datasets, that is, the warehouse should preserve information about the source (provenance) of warehouse entries so that users can determine exactly what proteins are part of the Swiss-Prot dataset or the PIR dataset. (2) Swiss-Prot and PIR might provide different information about the same protein, either because they disagree about the biological facts, or because they provide different commentary. (3) It simplifies the loaders, which need not be concerned with detecting and removing redundancy between different datasets. (4) It allows later execution of algorithms for computing nonredundant protein datasets, where different algorithms can be appropriate for different applications. A nonredundant protein DB could be created as a separate dataset within the warehouse that resulted from applying a redundancy-reduction algorithm to the Swiss-Prot and PIR datasets. This approach satisfies those users who will want to study Swiss-Prot per se, and those users who will want to work with a large nonredundant protein DB.

Each dataset is described by a row within the DataSet table, and contains attributes such as the dataset name, version number, release date, and reference URLs.

Our schema design also allows multiple versions of a given dataset to be loaded simultaneously, thus supporting queries that study the relationships among different versions of a dataset (such as: Is the error rate of Swiss-Prot increasing or decreasing? At what rate is the number of ORFs in the bacterial genomes within Swiss-Prot decreasing over time?). Having access to multiple versions of a DB simultaneously is also important when a newer DB version has changed in a way that interferes with important application software, or when one user is in the middle of a computational study that he wants to complete on the older DB version to maintain a consistent set of results, and another user of the same BioWarehouse instance wants to access the newest version of Swiss-Prot. Our approach gives warehouse administrators the option of deleting the old version of a DB and loading its new version, or loading the new version alongside the older one, allowing both to exist simultaneously.

This ability to maintain distinct datasets also enables users to capture locally produced data (e.g., protein-sequence data) in the warehouse by defining a separate warehouse dataset to hold these data.

Consider the fact that proteins, pathways, and other bioinformatics datatypes all need to cite literature references. We do not want the protein and pathway datatypes within BioWare-house to refer to different schema definitions of literature references, as would be done in warehousing approaches that create separate data-source-specific subschemas for every data source they include. We also do not want them to contain duplicate copies of the same definitions of literature references, which can happen with normalization schemes that blindly create new tables for every multivalued attribute of an entity. Therefore, we encode citations from all BioWarehouse datatypes and datasets within a single citation subschema within BioWarehouse. DB links are treated in a similar fashion – the subschema for each warehouse datatype encodes links to other DB objects in the same way.

Note that the current version of the warehouse schema is more oriented toward prokaryotes than eukaryotes; over time better support for eukaryotes is being added. This orientation applies to representation of genes, for example, the schema does not currently represent introns or alternative splicing.

### BioWarehouse schema implementation

**Figure 2:**
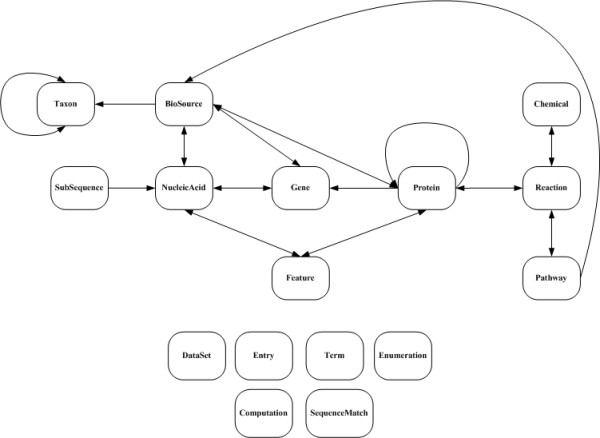
**The main datatypes in the BioWarehouse schema, and the relationships between them.** An arc is shown connecting two datatypes A and B if datatype A contains a column that references datatype B.

BioWarehouse supports several bioinformatics datatypes, each of which is implemented as one or more tables in the schema. The full schema is provided as supplementary material to this article as an ER diagram [see Additional file 1] to provide an overview of its organization, and as an SQL definition for readers interested in its details [see Additional file 2]. Figure 2 shows the main datatypes in the BioWarehouse schema, and the relationships between them. From left to right within this figure:

• Taxon: Describes taxonomic groups such as genus or species. Taxa are related to one another to represent the various hierarchical levels of taxonomic classification.

• BioSource: Represents the source of a biological material, whether nucleic acid molecule, protein, or gene. In addition to specifying the taxon of the material by cross-referencing to entries in the Taxon table, BioSource also stores source descriptors such as cell type, tissue, and sex of the organism. The loaders provided with the BioWarehouse all reference the NCBI Taxonomic DB [19] when creating entries in BioSource to provide a normalized set of taxonomic classifications.

• Nucleic Acid: Defines a DNA or RNA molecule, or a fragment thereof. A single contiguously sequenced fragment of a larger nucleic acid is represented by an entry in table SubSequence.

• Gene: BioWarehouse uses the prokaryotic notion of gene – a region of DNA that codes for a protein or RNA product, beginning with the transcriptional start site. Genes are related to entries in the NucleicAcid and Subsequence tables that define their sequence, and to their protein products. A gene may also be directly related to a BioSource.

• Protein: Defines proteins, including their amino acid sequences. Proteins are related to the genes that encode them, to the reactions they catalyze, and to features defined on their sequence. A protein may also be directly related to a BioSource. The schema can capture the subunit composition of a multimer, and does not require that a protein sequence be present.

• Feature: A subsequence of interest on an amino acid or nucleic acid sequence, such as a catalytic site, a phosphorylation site, a promoter region, or a transcription-factor binding site.

• Reaction: A spontaneous or catalyzed chemical reaction. A reaction is related to the protein that catalyzes it; to pathways that contain it; and to its chemical substrates, which can be small molecules or macromolecules such as proteins.

• Chemical: A small-molecular-weight chemical compound.

• Pathway: An ordered collection of biochemical reactions.

The bottom of Figure 2 lists some additional tables within the schema. Every entity within the warehouse (e.g., every nucleic acid, protein, and gene) has a row in the Entry table that defines metadata such as the time it was inserted in the warehouse, and its time of last update. Every warehouse entity is also associated with the dataset (DB version) from which it was derived; datasets themselves are defined in the DataSet table.

The Term table implements storage of external controlled vocabularies such as those provided by an ontology. Each entry in the Term table is a single term in a controlled vocabulary. The Enumeration table implements controlled vocabularies internal to BioWarehouse itself. For example, the BioWarehouse table BioSource contains a column "Sex" that is allowed to take on several possible controlled values including "Male" and "Female." The Enumeration table specifies the allowed controlled values for specified BioWarehouse tables and columns, making BioWarehouse self-documenting.

In addition to the preceding bioinformatics datatypes, the BioWarehouse represents many relationships among these datatypes. If the relationship is one-to-one, a column in the corresponding table simply contains the WID of the associated object. For example, a gene is associated with its nucleic acid in this manner. Relationships of higher multiplicity, such as many-to-many relationships, are implemented as linking tables that associate two or more primitives, such as a gene and a protein. A linking table contains WIDs for each of the related objects. This permits efficient, normalized representation of these relationships.

The warehouse also contains tables that implement auxiliary information, including descriptions of the source dataset of each warehouse entry, literature citations, human- and software-generated comments, DB cross-references (both internal and external to the warehouse), synonyms for named objects, and controlled vocabularies.

The BioWarehouse schema will evolve as new loaders are added to support new datatypes. SRI encourages other groups to submit new loader implementations, Java library extensions, and schema extensions to this open-source project. We do feel it is necessary for SRI to be the final arbiter of such extensions to ensure that the schema implementation remains compatible with its design principles to ensure continued maintainability and scalability of BioWarehouse. New versions of the schema will sometimes be incompatible with old versions, which will require loader modifications, and data reloading. Since public data sources must in any event be reloaded at regular intervals, the requirement of data reloading is not a large burden.

To support multiple DBMSs and their different flavors of SQL, each DBMS-specific schema is generated from a common schema template. The schema template consists of a framework using SQL syntax that is common among all supported DBMSs, interspersed with variables. Macro substitution converts the common schema to one that is conformant to the DBMS. The most common substitution is for primitive data types, which differ significantly across DBMSs.

### DB loaders

It is the responsibility of each loader to translate the flat file representation of its source DB into the warehouse schema. Typically, many of the source DB attributes are copied into the warehouse either verbatim or with minor transformations (e.g., converting "YES" and "NO" to "T" and "F"). The few source attributes not represented in the warehouse are generally ignored, although some attributes are added to the warehouse CommentTable. An example of warehouse translation semantics is shown in Table 1 for one file from the BioCyc collection of DBs.

**Table 1:** Semantics for translating the flatfile representation of a BioCyc reaction to columns of the BioWarehouse schema. The left column indicates source data from BioCyc; the right column indicates that data is transferred into the BioWarehouse. One row is added to the Reaction table for each flatfile reaction; rows to other tables are added as indicated. Table.Column indicates a column of a schema table. Attributes followed by [*] may occur multiple times per record. Note that some tables in this figure may not appear in Figure 2 because that figure is an abstraction of the full schema.

**BioCyc Attribute**	**Warehouse Semantics**
LEFT [*]	A row is added to A row is added to table Reactant. Value should match a Chemical.Name; its WID is stored as Product .WID. If a COEFFICIENT follows immediately, its value is stored as Reactant.Coefficient. Otherwise the value 1 is stored.
RIGHT [*]	A row is added to table product. Value should match a chemical.Name; its WID is stored as product .WID. If a COEFFICIENT follows immediately, its value is stored as Product .Coefficient. Otherwise the value 1 is stored.
COEFFICIENT	Reactant.Coefficient or Product.Coefficient for the immediately preceding LEFT or RIGHT attribute resp.
COMMON-NAME	A row is added to SynonymTable where SynonymTable.Syn is this value and SynonymTable. OtherWID is the WID of this reaction.
DELTAG0	Reaction.DeltaG
EC-NUMBER	Reaction.ECNumber
SPONTANEOUS?	Reaction.Spontaneous
SYNONYMS [*]	A row is added to SynonymTable where SynonymTable. Syn is this value and SynonymTable.OtherWID is the WID of this reaction
UNIQUE-ID	A row is added to DBID where DBID.XID is this value. and DBID.OtherWID is the WID of this reaction.

Loaders have been implemented in both the C and Java languages. C-based MySQL loaders interface with MySQL using the C API provided as part of MySQL. C-based Oracle loaders interface with Oracle using the Oracle Pro-C precompiler. Java-based loaders use the Java Database Connectivity (JDBC) API to interface with the DBMS. Each of these APIs allows SQL to be embedded and/or generated within its source language.

Loaders have been implemented for these bioinformatics DBs: UniProt (Swiss-Prot and TrEMBL [20]), ENZYME [21], Kyoto Encyclopedia of Genes and Genomes (KEGG) [22], the BioCyc collection of pathway/genome DBs (see URL [23-25]), the NCBI Taxonomy DB [19], GenBank [26], the Comprehensive Microbial Resource (CMR) [27], and Gene Ontology [28].

The architecture required to load datasets into a warehouse instance is akin to the process of compilation, but with the source code being a dataset and the object code being SQL insert statements to add the contents of the dataset to the warehouse. Standard parser generation tools (ANTLR for Java, Flex and Bison for C) are used throughout to specify the syntax of the input files, and associated loader actions.

A set of support routines is provided to create and manipulate an internal representation of the warehouse objects, including assignment of WIDs to objects, and the resolution of intra-dataset cross-references into WIDs. Loaders may make SQL queries to the local warehouse. SQL generation is performed by translating the internal representation into SQL statements.

Each loader has an associated manual describing its operations and any limitations in the loader. For example, the GenBank loader is currently recommended for use on prokaryotic sequences only.

### BioWarehouse java utility classes

The BioWarehouse implementation provides a set of Java classes with general utilities for interacting with BioWarehouse. These classes are useful for developers who want to construct new BioWarehouse loaders or applications. The classes provide methods for connecting to the database and for loading data into a BioWarehouse instance. These classes are packaged as a single module (a Java JAR file) that can be used by any Java-based loader or application. To abstract underlying DB specifics from the developer, a client application uses a factory class to obtain an instance of a Java DB class (Oracle or MySQL). The DB class provides methods for connecting to the database, performing queries, or doing BioWarehouse-specific tasks, such as obtaining a WID in an object-oriented, DBMS-independent way.

Similarly, we provide Java classes enabling a client to interact with the BioWarehouse tables in an object-oriented manner. The table classes are defined by an extensible class hierarchy that defines one class per table. The hierarchy greatly simplifies implementation of new table classes. Similar table types (e.g., all object tables or all linking tables) have their common functionality factored out into a base class. Each class provides accessors with methods for each property (column) of the table. These classes enable developers to create instances of the table classes (representing a single row in the table), set properties of the table object (corresponding to inserting values into the table), and store the data into the database. Methods are also provided to retrieve a row from a table given its primary key(s) (e.g., given a WID for object table entries), and to update or delete that row. Table 2 compares the processes for the SQL versus the Java method. In these examples, the Protein.DataSetWID is 2 and the Protein. WID is 5.

**Table 2:** Example BioWarehouse operations, implemented as SQL, and as operations in the Java utilities.

**Example**	**SQL**	**Java**
Create a new entry in the Protein table and add a comment for it in the CommentTable.	// Obtain a new WID SELECT WID_sequence.NextVal FROM dual INSERT INTO Protein (WID, Name, AASequence, DataSetWID) VALUES ('5', 'Sample Name', 'XXX', '2'); INSERT INTO CommentTable (OtherWID, Comm) VALUES ('5', 'Tester"s comment');	// A new WID is automatically obtained by the Protein class Protein protein = new Protein (2); protein. setName("Sample Name"); protein. set AASequence("XXX"); protein. addComment ("Tester's comment"); protein. storeQ;
Retrieve an existing entry in the Protein table and alter some of its data.	SELECT * FROM Protein WHERE WID = '5'; UPDATE Protein SET Name = 'New Name' WHERE WID = '5';	Protein protein = new Protein (2, 5); protein. load(); protein. setName("New Name""); protein. update();
Delete the Protein entry.	DELETE FROM Protein WHERE WID = '5';	Protein protein = new Protein (2, 5); Protein. delete ();

In addition to providing a convenient object-oriented interface to the BioWarehouse schema, the Java schema classes leverage the metadata capabilities of JDBC to perform data checking before inserting data. For example, we can detect if a text length exceeds the allowed maximum for a column before attempting an insert (which would otherwise fail). Another benefit is that we can perform unit tests on the classes to ensure compatibility with the current implementation of the BioWarehouse schema, and to confirm that all functions work as expected with each type of DBMS.

### Publichouse: Publicly queryable BioWarehouse server

As a convenience to users who do not want to maintain their own local BioWarehouse instance, SRI provides a publicly queryable BioWarehouse instance called Publichouse, which stores the open BioCyc, NCBI Taxonomy, ENZYME, and CMR DBs. Users can query Publichouse via Internet SQL queries. Because Publichouse stores only those DBs that their creators make openly available, users wanting to query other BioWarehouse-supported DBs must load those DBs into their own local BioWarehouse instance. That is, SRI cannot typically redistribute DBs that are not openly available, but most users will be able to download and install those DBs for their own local use.

Currently, Publichouse contains BioCyc DBs for ten organisms. However, in the near future we expect that number to increase to more than 150 organisms due to a joint effort between SRI and the Computational Genomics Group at the European Bioinformatics Institute to generate Pathway/Genome DBs for every completely sequenced bacterial and eukaryotic genome.

### User support and documentation

Extensive documentation is available for BioWarehouse within the software distribution. The available documentation is listed in a table of contents within the distribution at . The BioWarehouse documentation set includes release notes, a quick start guide, environment setup documentation, schema description and DBMS setup instructions, a description of the integration with the Dashboard for the February 2004 Bio-SPICE demonstration, and descriptions of Perl utilities and Perl demo scripts.

The table of contents also has a table listing statistics about each loader (latest supported version of its DB, last input DB size, load time, etc.) For each loader, there are two pieces of documentation: how to build and run the loader, and a manual for developers describing the details of the loader implementation and mappings from the source DB schema to the BioWarehouse schema.

Bug reports and requests for assistance should be sent to support@biowarehouse.org.

## Results and discussion

Here we present results obtained by BioWarehouse in its use by several bioinformatics projects, and a performance analysis of BioWarehouse.

An SRI project is developing algorithms for predicting which genes within a sequenced genome code for missing enzymes within metabolic pathways predicted for that genome [29]. BioWarehouse fills several roles within that project: it is used to construct a complete and nonredundant dataset of sequenced enzymes by combining protein sequences from the UniProt and PIR DBs, and by removing from the resulting dataset those sequences that share a specified level of sequence similarity. Our current research involves extending the pathway hole filling algorithm with information from genome-context methods such as phylogenetic signatures, which is obtained from BioWarehouse thanks to the large all-against-all BLAST results stored within CMR.

Another SRI project is comparing the data content of the EcoCyc and KEGG DBs using BioWarehouse to access the KEGG data in a computable form. Jeremy Zucker of Harvard is using BioWarehouse as a component of an automated pipeline that will construct metabolic flux models from annotated genomes [30] as part of the Bio-SPICE project [31]. Zucker is also using BioWarehouse to develop a translator from KEGG to the BioPAX pathway exchange standard (see URL [32]).

### Enzyme sequence completeness

An SRI project is using BioWarehouse to determine the completeness with which biochemically characterized enzymes have been sequenced. Specifically, we answered the question: in the Swiss-Prot, TrEMBL, PIR, CMR, or BioCyc DBs, what fraction of enzymes that have been assigned EC numbers are associated with at least one protein sequence [33]? Illustrated here is a type of problem that can be solved with BioWarehouse. This problem is not a systematic or formal evaluation of BioWarehouse.

This question is significant since the identification of an enzyme in a newly sequenced genome cannot be made if no sequence is known for an enzyme with that activity. Unrecognizable enzymes therefore limit the completeness of genome annotations and of metabolic pathway predictions. Furthermore, we cannot genetically engineer an unsequenced enzyme into a new organism to accomplish a metabolic engineering goal, because we do not know which gene to insert to provide the needed enzyme activity.

EC numbers (Enzyme Commission numbers) constitute a classification system for enzyme function developed over the course of many years by the Nomenclature Committee of the International Union of Biochemistry and Molecular Biology (NC-IUBMB) [34] (see also URL [35]). The classification system is four levels deep, and each enzyme function at a leaf of the classification tree is assigned a unique tuple of four numbers. The enzyme functions are classified according to the chemical transformation of the enzymatic reaction. For example, the enzyme "tryptophan synthase" is assigned the EC number 4.2.1.20, with class "4" indicating that this enzyme belongs to the class of lyase enzymes.

The ENZYME DB is an electronic version of the EC system. Version 33.0 of ENZYME (all DB versions used to address this question were those of December 2003 except for CMR) contains 4208 distinct EC numbers, of which 472 have been deleted or transferred to new numbers; it therefore lists 3,736 different biochemically characterized enzyme activities. Warehouse queries allowed us to determine the distinct EC number content of each DB as shown in Table 3. We provide execution times to give the reader a sense of how fast these queries execute on BioWarehouse.

**Table 3:** EC number content of Swiss-Prot, TrEMBL, PIR, CMR, and BioCyc. **Total EC numbers **is the number of distinct EC numbers in each DB. **Incremental Novel ECnumbers **is the number of EC numbers present in a given DB that were not present in all preceding DBs in the table. For example, 158 EC numbers are present in PIR that are not present in either Swiss-Prot or TrEMBL. We do not show all pairwise combinations of shared EC numbers between DBs because it is not particularly meaningful. **Time **is the execution time of the SQL query listed below to compute the distinct EC numbers in each DB on a dual-CPU 1 GHz Pentium Oracle server with 2 GB memory running Linux. Note that because a PIR warehouse loader does not exist at this time, PIR statistics were obtained through queries to an XML version of PIR outside the warehouse. Note also that it is somewhat surprising that PIR contains EC numbers not found in Swiss-Prot, because version 42.6 is a UniProt version of Swiss-Prot that incorporates data from PIR. It appears that not all data from PIR entries has been incorporated into UniProt.

**Database**	**Version**	**Total EC numbers**	**Incremental Novel EC numbers**	**Time**
Swiss-Prot	42.6	1899	1899	7.6 s
TrEMBL	25.4	1678	316	53 s
PIR	PIR-PSD 78.03	1695	108	na
CMR	April, 2003	1230	26	159 s
BioCyc	7.6	1357	44	2.6 s

In total, these DBs reference 2380 distinct EC numbers, or 64% of all known EC numbers. Therefore, for 1356 EC numbers (36%), no sequence is known. We refer to such EC numbers as "orphan activities."

Two qualifications to the preceding analysis should be stated. First, the EC system is incomplete in that it does not yet include a number of enzymes whose biochemical activities have been characterized. The MetaCyc DB alone describes 890 enzyme activities that have no associated EC number. The true number of biochemically characterized enzymes is probably between 5000 and 6000.

Second, there could be entries in UniProt that omit EC number annotations, which, if properly annotated, would provide sequences for some of these enzymes. We have performed manual literature searches and DB searches in Swiss-Prot and TrEMBL for 228 orphan activities, and have found sequences for 18% of them. Additional information about this survey can be found at [36].

We conclude from this analysis that an Enzyme Genomics project should be initiated with the goal of obtaining at least one sequence for each unsequenced enzyme activity. Such a project would boost the accuracy of genome annotation at the level of both proteins and metabolic pathways, and would remove barriers to metabolic engineering.

One SQL query that implements the analysis in Table 3 is shown below. It queries a single column of the Reaction table for a set of EC number values. Care is taken to filter out partial EC numbers and multiple EC numbers by using query expressions containing wildcards (percent signs). Partial EC numbers contain a hyphen in place of one or more of its numbers (for example, 4.2.1.-). Occasionally, multiple EC numbers will be associated with a reaction. These are separated by forward slashes.

select distinct ecnumber from Reaction

where datasetwid = [DATABASE-WID]

and ecnumber not like '%-%'

### Performance analysis

We present the run times of the following queries to illustrate the performance of BioWare-house using the hardware described in Table 3. All queries were run against an Oracle BioWarehouse instance running on a dual-processor 2.66 GHz Pentium 4 processor machine with 2 GB of memory. The instance contained all seven DBs loaded, which occupies approximately 10 GB. Additional queries are provided in supplementary material [see Additional file 3]. Our intention is not to provide a thorough performance analysis, but simply to show that even with a number of large datasets loaded, simple queries are evaluated quickly.

Query 1: "select * from Protein where name = 'Zyxin' and DataSetWID = (select WID from DataSet where Name = 'Swiss-Prot')"

Query 1 retrieves three proteins (including their sequences) from Swiss-Prot by name in 204 milliseconds. The Protein table contains 1694037 rows.

Query 2: "select AASequence from Protein where WID = (select OtherWID from DBID where XID like ' ZYX\_MOUSE%') and DataSetWID = (select WID from DataSet where Name = 'Swiss-Prot')"

Query 2 retrieves the sequence of one protein from Swiss-Prot given its Swiss-Prot ID in 114 milliseconds. The Protein table contains 1694037 rows. The DBID table contains 3685779 rows.

### Related work

BioWarehouse is distinguished from other bioinformatics DB warehouse efforts in the following respects. BioWarehouse supports a unique collection of bioinformatics data sources that are not supported by any other warehousing system, with a unique focus on integrating metabolic pathway and enzyme databases, and on integrating multiple sources of information on completely sequenced microbial genomes. BioWarehouse offers a unique approach to scalability that will allow it to scale to a large number of data sources; that approach combines a methodology for limiting the growth of schema size and complexity, which are critical attributes of warehousing systems; with an open-source development model that will allow other groups to contribute new loaders to BioWarehouse; and a Java library that simplifies the process of writing new loaders. BioWarehouse runs on two industry standard DBMSs – Oracle and MySQL – whereas some other warehouses run on non-standard DBMSs, or on only one DBMS.

Ritter created a warehouse of several bioinformatics DBs called IGD [37,38] using AceDB [39] as the underlying DBMS. AceDB did not have adequate scalability for the handful of DBs that Ritter integrated. The AceDB DBMS has other limitations as well, such as its lack of a well-crafted and standardized query language.

SRS [40] uses a variant of the warehouse approach that is highly text oriented rather than oriented toward structured data values, meaning that the ability of SRS to compute with its data is constrained. Furthermore, SRS has no integrated schema, meaning that it does not attempt to unify the disparate semantics of the DBs that it integrates. SRS is also a read-only system – it does not have standard DBMS update operations based on atomic transactions. Therefore, users cannot insert and update data from their own laboratories into an SRS DB by using transactions. Finally, SRS is not an industry-standard DBMS, and does not support a full-featured complex-query language. The proprietary nature of SRS makes its workings very difficult to discern.

The GUS [11] project at the University of Pennsylvania has somewhat different goals than BioWarehouse. GUS is not designed for integration of public bioinformatics DBs, but is oriented toward implementation of bioinformatics applications that require integration of custom local data collections. Therefore, GUS does not provide loader tools for public bioinformatics databases. GUS emphasizes issues of data provenance and of detecting changes in the underlying data sources. GUS is implemented for the Oracle and Postgresql DBMSs. GUS has an extremely large schema (approximately 480 tables – see [41]) that is likely to limit its understandability by other groups and therefore their ability to query and extend GUS. GUS provides a publicly queryable DBMS server.

The Atlas warehouse system [42] is remarkably similar to BioWarehouse in its design. Like BioWarehouse, Atlas uses a set of loaders to transform data from source-DB files into a relational DBMS schema that models several bioinformatics datatypes. Atlas focuses integrating data on biological sequences, species taxonomies, molecular interactions, gene annotations, and ontologies. Atlas and BioWarehouse support four DBs in common: GenBank, UniProt, Gene Ontology, and NCBI Taxonomy. Atlas runs on MySQL only, and has a medium-sized schema of approximately 70 tables, which according to the Atlas schema diagram in [42] are not shared among different biological datatypes.

EnsMart [43] is another warehouse-based system that is distinguished by its user-friendly query front end that allows users to compose complex queries interactively. EnsMart runs on Oracle and MySQL. Datatypes currently supported by EnsMart are genes, SNP data, and controlled vocabularies. The size and full design principles of the schema underlying EnsMart (and its parent BioMart) are unclear. Because the number of datatypes supported is small, so is the schema, and it is unclear how well the schema will scale as more datatypes are added. One schema design principle stated is the use of the star-schema approach, which is known to produce schemas that have large numbers of tables for each datatype, and thus could prove troublesome for scalability.

The Biozon system (see URL [44]) is also warehouse-based, and also provides a user-friendly Web interface for constructing complex queries.

## Conclusion

We have presented the design and implementation of the open source BioWarehouse toolkit for constructing bioinformatics DB warehouses. BioWarehouse consists of a global relational DB schema for important bioinformatics datatypes, and a set of loader tools that parse public bioinformatics DBs and load their contents into that schema. BioWarehouse has been implemented for both the Oracle and MySQL relational DBMSs. The toolkit can be downloaded and installed by users who want to configure their own warehouses on local hardware. In addition, SRI operates a BioWarehouse instance called Publichouse that contains the Bio-Cyc, NCBI Taxonomy, ENZYME, and CMR DBs, and can be queried by the public. The utility of BioWarehouse has been proven as a result of its use in several research projects at SRI and elsewhere.

## Availability and requirements

• **Project name: **BioWarehouse

• **Project home page: **

• **Operating system(s): **Linux

• **Programming languages: **C and Java

• **License: **Mozilla

• **Any restrictions to use by non-academics: **None

## Authors' contributions

TL, YP, VW, PG, DSC, and JT were involved in implementing one or more loaders. All authors contributed to the system design of BioWarehouse, and to the design of its schema. PK supervised the project. PK drafted the manuscript; other authors wrote sections of the manuscript. All authors read and approved the final manuscript.

## Supplementary Material

Additional file 1 BioWarehouse Schema ER Diagram; An entity-relationship diagram for the BioWarehouse schema. Click here for file

Additional file 2 BioWarehouse Schema Definition; An SQL definition of the BioWarehouse schema. Click here for file

Additional file 3 Additional Queries and Timings; Additional example queries to Bio Warehouse and the time they required to execute. Click here for file
