# Supplementary figures and images for: BioWarehouse: a bioinformatics database warehouse toolkit
Source: BMC Bioinformatics. 2006 Mar 23;7:170. doi: 10.1186/1471-2105-7-170 (PMC1444936; doi:10.1186/1471-2105-7-170)

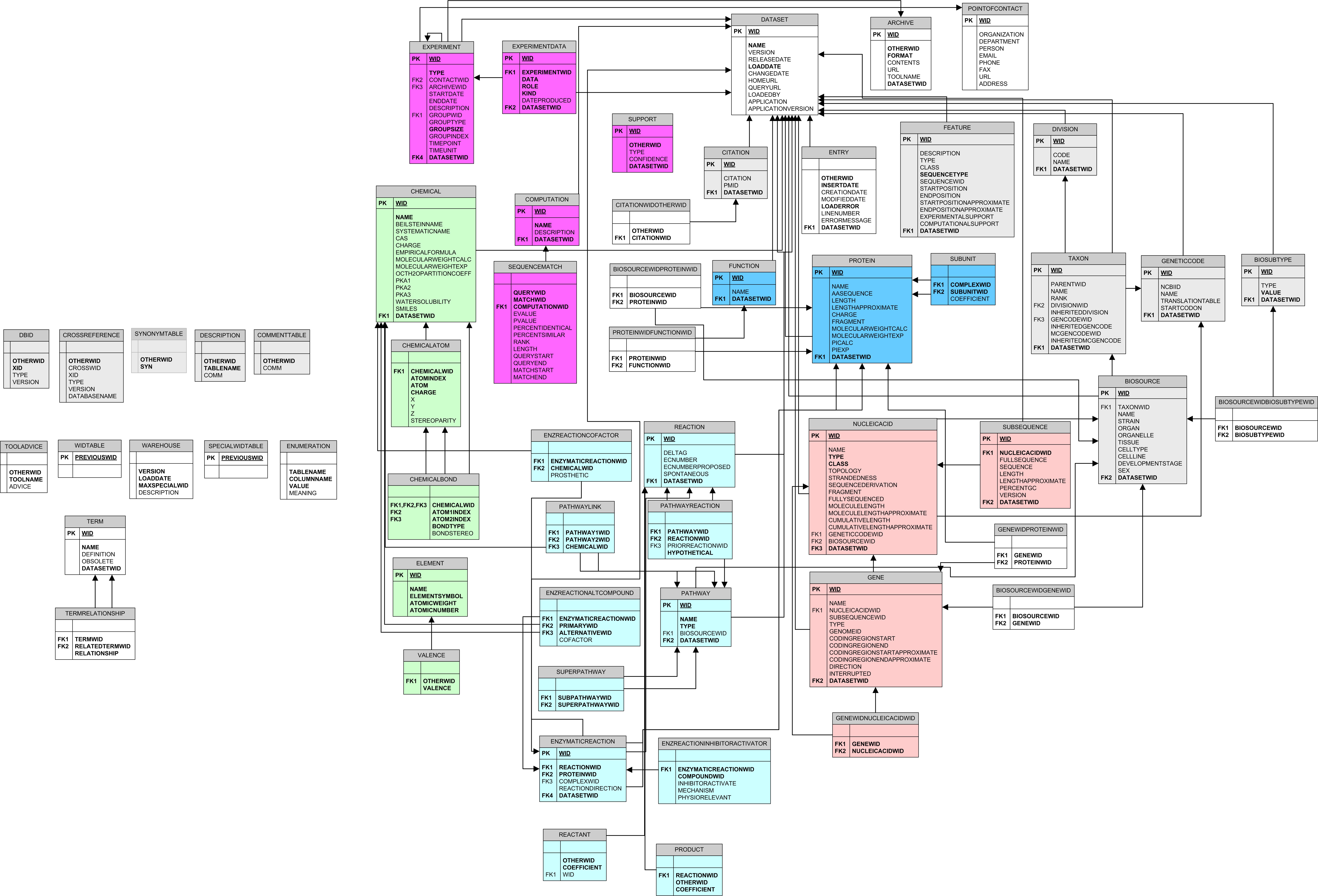

Supplement: Additional file 1 — BioWarehouse Schema ER Diagram; An entity-relationship diagram for the BioWarehouse schema. [file 1471-2105-7-170-S1.jpeg]
